# Characteristics and Prognostic Impact of Pneumonitis during Systemic Anti-Cancer Therapy in Patients with Advanced Non-Small-Cell Lung Cancer

**DOI:** 10.1371/journal.pone.0168465

**Published:** 2016-12-22

**Authors:** Daichi Fujimoto, Ryoji Kato, Takeshi Morimoto, Ryoko Shimizu, Yuki Sato, Mariko Kogo, Jiro Ito, Shunsuke Teraoka, Kazuma Nagata, Atsushi Nakagawa, Kojiro Otsuka, Keisuke Tomii

**Affiliations:** 1 Department of Respiratory Medicine, Kobe City Medical Center General Hospital, Kobe, Japan; 2 Clinical Research Center, Kobe City Medical Center General Hospital, Kobe, Japan; 3 Department of Clinical Epidemiology, Hyogo College of Medicine, Nishinomiya, Japan; Seoul National University College of Pharmacy, REPUBLIC OF KOREA

## Abstract

**Background:**

Data on characteristics, outcomes, and prognosis of advanced non-small-cell lung cancer (NSCLC) patients who develop pneumonitis during systemic anti-cancer therapy (pneumonitis) are currently lacking.

**Methods:**

We conducted a retrospective cohort study of 910 consecutive patients diagnosed with advanced NSCLC between January 2004 and January 2014. Of these, 140 patients were excluded because they did not receive systemic anti-cancer therapy at this hospital.

**Results:**

A total of 770 patients were included in the study, of whom 44 (6%) were diagnosed with pneumonitis. The mortality rate of pneumonitis was 36%. The incidence of pneumonitis was independently associated with pre-existing ILD (adjusted odds ratio, 2.99, P = 0.008), and survivors were significantly associated with younger age (P = 0.003) and radiographic non-acute interstitial pneumonia pattern (P = 0.004). In all patients, pneumonitis was identified as an independent predictor of overall survival (OS) (adjusted hazard ratio 1.53, 95% CI, 1.09–2.09, P = 0.015). Performance status was poor in 82% of survivors of pneumonitis; in 62% of survivors, the PS worsened after the pneumonitis improved. Additionally, 54% of survivors received no further systemic anti-cancer therapy after pneumonitis. The median survival time of survivors after pneumonitis was 3.5 months (95% CI, 2.3–7.2 months).

**Conclusions:**

Our study indicated that 6% of patients with advanced NSCLC developed pneumonitis during systemic anti-cancer therapy. The early mortality rate of pneumonitis is high, and the survival and PS after pneumonitis is extremely poor. Additionally, pneumonitis has an adverse impact on the survival of patients with advanced NSCLC. These data should be considered for the management of pneumonitis, and we recommend that future work focuses on pneumonitis particularly to improve the survival of patients with advanced NSCLC.

## Introduction

Lung cancer is the leading cause of cancer-related deaths worldwide [1, 2]. Non-small-cell lung cancer (NSCLC) accounts for approximately 80% of lung cancer cases, and the majority are at an advanced stage, that is, unresectable and metastatic upon their initial diagnosis. As systemic anti-cancer therapy is the main treatment option for these patients, some individuals develop interstitial lung disease (ILD) [1, 2]. Indeed, about 70% of severe pneumonitis cases are associated with systemic anti-cancer therapy, and the early mortality rate of these patients is high [3, 4]. Furthermore, pneumonitis during systemic anti-cancer therapy (pneumonitis) is the most frequent cause of systemic anti-cancer therapy-related mortality [5]. However, there are few data on pneumonitis, and most are in the form of case reports and review articles.

The clinical situation is quite different in patients with advanced NSCLC and pneumonitis, compared with those with pneumonitis without advanced cancer, because the prognosis of advanced NSCLC itself is generally poor [1]. Survival data and clinical characteristics of patients with pneumonitis and advanced NSCLC would be useful to assess the clinical importance of pneumonitis. Further knowledge of the mortality rate and the clinical course of pneumonitis can facilitate shared decision-making for the treatment of acute respiratory failure associated with pneumonitis in advanced NSCLC patients. Therefore, an understanding of pneumonitis in these subjects is important for improving the management not only of pneumonitis, but also advanced NSCLC. The aim of this study, therefore, is to understand the characteristics, outcomes, and survival of advanced NSCLC patients who developed pneumonitis.

## Patients and Methods

### Patients

**Fig 1:**
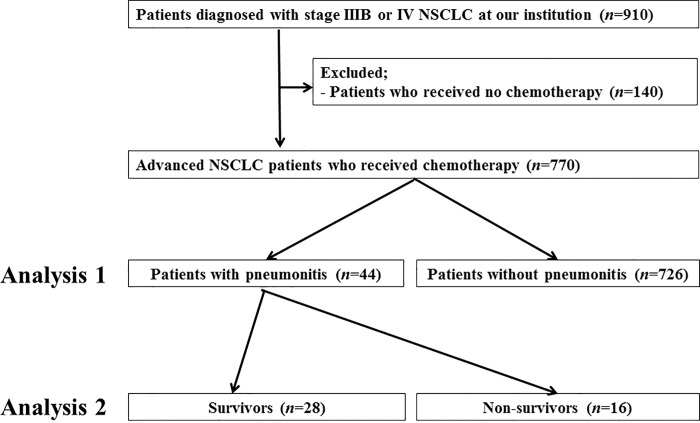
Patient selection and exclusion criteria. NSCLC: non-small cell lung cancer;

We conducted a retrospective cohort study at Kobe City Medical Center General Hospital, a tertiary referral centre. We identified 910 consecutive patients diagnosed with advanced (stage IIIB or IV) NSCLC at this hospital between January 2004 and January 2014. Of these, 140 patients were excluded as they did not receive systemic anti-cancer therapy at this hospital (Fig 1). Patients who reported never having smoked were defined as never smokers, those who had smoked within 1 year of the diagnosis were categorized as current smokers and the rest were considered former smokers. The clinical stage of all patients was established according to the TNM classification, 7th edition [6]. Overall survival (OS) was measured as the period from the diagnosis of NSCLC until death from any cause or the end of the follow-up period. Worsening of performance status (PS) was defined as change from PS 0–1 to PS 2–4, or from PS 2 to a PS 3–4 after pneumonitis improvement. Pre-existing ILD was diagnosed based on clinical features and results of the pre-treatment high-resolution computed tomography (HRCT) of the chest. All patients underwent HRCT as part of routine clinical practice, and the presence of ILD was evaluated by at least two pulmonologists who were unaware of the patients’ clinical status. Pre-existing ILD was defined as ILD before the first-line therapy. We classified the radiographic HRCT images of ILD into usual interstitial pneumonia (UIP) and non-UIP patterns. The diagnosis of a UIP pattern was determined using CT features defined by the International Consensus Statement of the American Thoracic Society and European Respiratory Society [7]. The UIP and possible UIP patterns were all considered UIP patterns in this study. We isolated tumor DNA from various specimens and analysed epidermal growth factor receptor gene (*EGFR*) mutation status at exons 18–21 using the peptide nucleic acid-locked nucleic acid polymerase chain reaction clamp method, as described previously [8]. The study was approved by the Ethics Committee of Kobe City Medical Center General Hospital.

### Pneumonitis during systemic anti-cancer therapy (pneumonitis)

Pneumonitis was defined using the following criteria, which were based on the criteria proposed by the Idiopathic Pulmonary Fibrosis Clinical Research Network [9] and the definition used in a recent randomized control trial [10]: 1) unexplained worsening of dyspnea within the past 30 days; 2) high resolution computed tomography (HRCT) with new bilateral ground-glass opacity or consolidation; 3) no evidence of pulmonary infection by bronchoalveolar lavage (BAL), endotracheal aspiration or sputum culture, in combination with negative blood tests for other potentially infectious pathogens (e.g., *Pneumocystis jirovecii*, Cytomegalovirus); 4) no evidence of malignant cells in BAL fluid to exclude lymphangitic spread of the cancer, 5) exclusion of left heart failure and other possible causes of acute respiratory failure [11]; 6) and less than a 4-week interval between the last administration of systemic anti-cancer therapy and the onset of a pulmonary shadow [12, 13]. Patients who developed acute ILD within 6 months after thoracic radiotherapy were not considered as having pneumonitis because the time for radiation pneumonitis onset is usually 6 months [14–16]. The pneumonitis cases were further divided into the following categories based on bronchoscopic test with bronchoalveolar lavage (BAL) [3, 4, 11, 17]: 1) possible pneumonitis, in which the diagnosis was based on physical examination, medical history, laboratory data, and HRCT scan images; 2) probable pneumonitis, in which the diagnosis was based on a bronchoscopic test with BAL in addition to the diagnostic examinations of possible pneumonitis mentioned above. Pneumonitis was categorised based on pneumonitis/pulmonary infiltrates by the National Cancer Institute Common Terminology Criteria version 4.0 as follows: Grade 2, symptomatic, medical intervention indicated, and limited instrumental activities of daily living; Grade 3, severe symptoms limiting self-care activities of daily living and oxygen indicated; Grade 4, life-threatening respiratory compromise and urgent intervention indicated; Grade 5, death. Patients with Grades 2–4 pneumonitis were defined as survivors. In each case, radiographic patterns of pneumonitis were classified according to previous reports, as 1) acute interstitial pneumonia (AIP) pattern, 2) non-specific interstitial pneumonia pattern, 3) organizing pneumonia pattern, 4) hypersensitivity pneumonitis (HP) pattern, and 5) unclassifiable pattern [18, 19].

### Ethics statement

This study was conducted with the approval of the Kobe City Medical Center General Hospital Ethics Committee. Patient information was anonymized and de-identified prior to analysis. The need for written informed consent was waived for individual participants. We announced this study on the Internet, and patients had the option of refusing study participation.

### Statistical analysis

Continuous variables were analysed using Student’s *t*-tests. Dichotomous variables were analysed using χ^2^ or Fisher’s exact tests, as appropriate. The Kaplan–Meier method was used to estimate the survival outcomes, and groups were compared using the log-rank test. Univariate and multivariate logistic regression models were developed to estimate odds ratios (ORs) and 95% confidence intervals (CIs). Multivariate logistic regression model was constructed on variables selected by stronger relationships in univariate analyses (P<0.05) because of the small number of events. Cox proportional hazard models were used to estimate the hazard ratios (HRs) for factors associated with survival in both univariate and multivariate analyses. Multivariate analysis was performed on all important clinical factors (age, sex, smoking status, ECOG PS, *EGFR* status, stage, type of first-line therapy, pre-existing ILD, and pneumonitis). The results are expressed as HRs with 95% CI. A P value of <0.05 was considered to indicate statistical significance. We conducted the statistical analyses using JMP 11 software (SAS Institute, Cary, NC, USA).

## Results

### Characteristics and survival of NSCLC patients (analysis 1)

In total, 770 patients with advanced NSCLC were included in the study. Of these, 44 (6%) were diagnosed with pneumonitis during systemic anti-cancer therapy (21 probable and 23 possible pneumonitis cases). There were 16, six, 17, and five patients with pneumonitis Grades 5, 4, 3, and 2, respectively. Patient characteristics and comparisons between pneumonitis and non-pneumonitis patients are summarised in Table 1. Most patients (77%) had a PS of 0 or 1. *EGFR* mutations were investigated in 463 patients (60%) and were detected in 174 patients (38%). Sixty-nine patients (9%) had pre-existing ILD.

**Table 1:** Characteristics of and comparison between patients with and without pneumonitis during systemic anti-cancer therapy

Patient characteristics	Total (%)	Pneumonitis	Non-pneumonitis	P-value
		(%)	(%)	
	(N = 770)	(N = 44)	(N = 726)	
Age (years), mean (SD)	67.8 (10.7)	69.7 (8.5)	67.7 (10.8)	0.217
Sex				
Male	506 (66)	35 (80)	471 (65)	0.047
Smoking status				
Never-smoker	240 (31)	7 (16)	233 (32)	0.024
Histology				
Adenocarcinoma	564 (73)	32 (73)	532 (73)	0.936*
Squamous	174 (23)	9 (20)	165 (23)	
NSCLC-NOS	30 (4)	1 (2)	29 (4)	
Other	2 (0)	2 (5)	0 (0)	
ECOG PS				
0 or 1	594 (77)	34 (77)	560 (77)	0.983
2–4	176 (23)	10 (23)	166 (23)	
Stage				
III B	154 (20)	11 (25)	143 (20)	0.393
IV	616 (80)	33 (75)	583 (80)	
*EGFR* status				
Mutated	174 (23)	4 (9)	170 (23)	0.027*
Wild-type	289 (38)	18 (41)	271 (37)	
Not investigated	307 (39)	22 (50)	285 (40)	
Pre-existing ILD	69 (9)	11 (25)	58 (8)	<0.001
UIP pattern	47	6	41	0.303
Non-UIP pattern	22	5	17	
Thoracic radiotherapy	94 (12)	4 (9)	90 (12)	0.641
First-line therapy				
Molecular-targeted therapy (TKIs)	140 (18)	6 (14)	134 (18)	
EGFR-TKIs	138	6	132	
Crizotinib	2	0	2	
Cytotoxic agents (combination **)	550 (72)	32 (72)	518 (71)	
Containing paclitaxel	168	7	161	
Containing vinorelbine	163	10	153	
Containing pemetrexed	73	2	71	
Containing gemcitabine	56	5	51	
Containing docetaxel	35	1	34	
Containing S1	28	3	25	
Containing etoposide	22	5	17	
Containing irinotecan	10	0	10	
Others	3	0	3	
Cytotoxic agents (monotherapy)	80 (10)	6 (14)	74 (11)	
Pemetrexed	24	2	22	
Paclitaxel	15	0	15	
S1	13	3	10	
Vinorelbine	9	0	9	
Gemcitabine	9	1	8	
Docetaxel	6	0	6	
Others	4	0	4	
Diagnosis of pneumonitis				
Probable		21 (48)		
Possible		23 (52)		
Pneumonitis grade				
Grade 5		16 (36)		
Grade 4		6 (14)		
Grade 3		17 (39)		
Grade 2		5 (11)		

SD, standard deviation; NSCLC, non-small-cell lung cancer; NOS, not otherwise specified; ECOG PS, Eastern Cooperative Oncology Group Performance Status; EGFR, epidermal growth factor receptor gene; ILD, interstitial lung disease; UIP, usual interstitial pneumonia; TKIs, tyrosine kinase inhibitors

*Patients with adenocarcinoma and non-adenocarcinoma, and patients with mutated and wild-type/not investigated EGFR were compared using χ2 tests.

** Eight patients received combination therapy other than platinum combination.

Comparison of the clinical profiles of pneumonitis and non-pneumonitis patients showed that pneumonitis patients included significantly higher proportions of current or former smokers (37/530 vs. 7/240, respectively, P = 0.024), patients with pre-existing ILD (11/69 vs. 33/701, respectively, P<0.001) and with wild-type or *EGFR* not assessed (40/596 vs. 4/174, respectively, P = 0.027). Fifteen patients developed pneumonitis during first-line therapy. Of these, patients with pneumonitis included significantly higher proportions of patients with pre-existing ILD (4/69 vs. 11/701, respectively, P = 0.038).

Multivariate logistic regression analysis showed that pre-existing ILD (OR, 2.99; 95% CI, 1.36–6.21; P = 0.008) was independently associated with pneumonitis. The results of this analysis are listed in Table 2.

**Table 2:** Factors associated with pneumonitis during systemic anti-cancer therapy (N = 770)

Variables	Multivariate analysis		
	OR	95% CI	P-value
Sex (female/male)	0.74	0.28–1.77	0.517
Smoking status (never-/current or former smoker)	0.70	0.24–1.91	0.508
*EGFR* mutations (yes/no)	0.49	0.14–1.32	0.167
Pre-existing ILD (yes/no)	2.99	1.36–6.21	0.008

OR, odds ratio; CI, confidence interval; EGFR, epidermal growth factor receptor gene; ILD, interstitial lung disease

**Fig 2:**
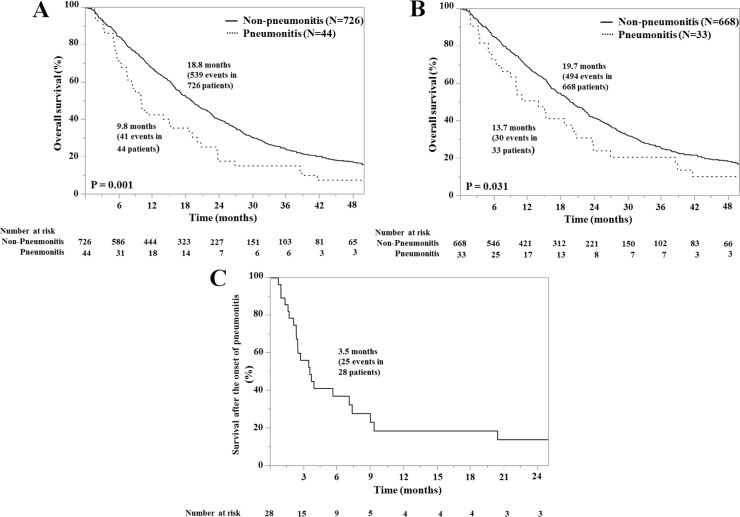
Kaplan–Meier overall survival curves after the diagnosis of NSCLC in all patients with or without pneumonitis during systemic anti-cancer therapy (pneumonitis) (Fig 2A) and those who did not have pre-existing ILD with or without pneumonitis (Fig 2B), and survival time after the onset of pneumonitis in survivors of pneumonitis (Fig 2C).

The OS time from the diagnosis of advanced NSCLC is presented in Fig 2A and summarised in Table 3. At the time of analysis, the median OS was 18.4 (95% CI, 16.7–20.0) months. Shorter OS was significantly associated with older age, male sex, current or former smokers, poor PS, wild-type EGFR or not assessed, pre-existing ILD, and pneumonitis. A multivariate Cox model showed that independent predictors of OS were older age (HR 1.25, 95% CI, 1.04–1.50, P = 0.019), poor PS (HR 2.47, 95% CI, 2.03–2.98, P<0.001), wild-type or not investigated EGFR (HR 2.08, 95% CI, 1.63–2.67, P<0.001), stage IV (HR 1.46, 95% CI, 1.18–1.82, P<0.001), pre-existing ILD (HR 1.90, 95% CI, 1.41–2.53, P<0.001), and pneumonitis (HR 1.53, 95% CI, 1.09–2.09, P = 0.015). We further investigated the prognostic impact of pneumonitis in NSCLC patients without pre-existing ILD (Fig 2B). Shorter OS was also significantly associated with pneumonitis in these patients.

**Table 3:** Analyses of overall survival time in all patients (N = 770)

Characteristics	No. of patients	Median survival	Univariate analysis		Multivariate analysis	
	(%)	(months)	HR (95% CI)	P-value	HR (95% CI)	P-value
**Total**						
Age				<0.001		0.019
≥75 years	219 (28)	15.1	1.46 (1.22–1.74)		1.25 (1.04–1.50)	
<75 years	551 (72)	20.0	referencereference		reference	
Sex				<0.001		0.076
Male	506 (66)	15.9	1.41 (1.19–1.68)		1.23 (0.98–1.55)	
Female	264 (34)	25.0	reference		reference	
Smoking status				0.007		0.692
Never-smoker	240 (31)	22.6	0.79 (0.66–0.94)		1.04 (0.83–1.33)	
Current or former smoker	530 (69)	15.9	reference		reference	
ECOG PS				<0.001		<0.001
ECOG 0,1	594 (77)	21.5	0.39 (0.32–0.47)		0.40 (0.33–0.49)	
ECOG 2–4	176 (23)	8.4	reference		reference	
*EGFR* mutations				<0.001		<0.001
Yes	174 (23)	28.7	0.54 (0.44–0.66)		0.48 (0.38–0.61)	
No or not investigated	596 (77)	15.4	reference		reference	
Stage				0.059		<0.001
IIIB	154 (20)	18.3	0.82 (0.66–1.01)		0.69 (0.55–0.85)	
IV	616 (80)	18.3	reference		reference	
Type of first-line therapy				0.518		0.106
TKIs	140 (18)	19.4	0.93 (0.74–1.15)		1.26 (0.95–1.64)	
Cytotoxic chemotherapy	630 (82)	18.3	reference		reference	
Pre-existing ILD				<0.001		<0.001
Yes	69 (9)	9.8	2.21 (1.65–2.90)		1.90 (1.41–2.52)	
No	701 (91)	19.3	reference		reference	
Pneumonitis during systemic anti-cancer therapy				0.003		0.015
Yes	44 (6)	9.8	1.68 (1.20–2.27)		1.53 (1.09–2.09)	
No	726 (94)	18.8	reference		reference	

ECOG PS, Eastern Cooperative Oncology Group Performance Status; EGFR, epidermal growth factor receptor gene; ILD, interstitial lung disease; HR, hazard ratio; CI, confidence interval; TKIs, tyrosine kinase inhibitors

### Survival of the pneumonitis patients (analysis 2)

Among patients with advanced NSCLC and pneumonitis, the median time between the diagnosis of advanced NSCLC and onset of pneumonitis was 6.0 (95% CI, 4.3–8.8) months. Most pneumonitis cases (66%) occurred during the second, third, or subsequent lines of systemic anti-cancer therapy. Additionally, the median time between the start of the first cycle of therapy that triggered pneumonitis and the onset of pneumonitis was 1.3 (95% CI, 0.9–1.7) months. Thirty-two patients (73%) developed pneumonitis during one or two cycles of therapy.

Patient characteristics and comparisons between survivors (Grade 2–4 pneumonitis patients) and non-survivors (Grade 5 pneumonitis patients) are shown in Table 4. Survivors were significantly associated with younger age (P = 0.003) and radiographic non-AIP pattern (P = 0.004). Sixteen patients (36%) died from respiratory failure within 2 to 30 days (median, 10 days) of pneumonitis onset. All of the pneumonitis patients received steroid therapy. Seven of eight pneumonitis patients died while on mechanical ventilation.

**Table 4:** Characteristics and outcomes of patients with pneumonitis during systemic anti-cancer therapy (N = 44)

Characteristics	Total (%)	Survivors	Non-survivors	P-value
	(N = 44)	(N = 28)	(N = 16)	
Age (years), mean (SD)	69.7 (8.5)	66.9 (7.4)	74.6 (8.3)	0.003
Sex				
Male	35 (80)	25 (89)	10 (62)	0.053
Smoking status				
Never-smoker	7 (16)	3 (11)	4 (25)	0.235
Pre-existing ILD	11 (25)	6 (21)	5 (31)	0.492
Stage				
IIIB	11 (25)	9 (32)	2 (13)	0.278
IV	33 (75)	19 (68)	14 (87)	
Number of chemotherapy line *				
1st	15 (34)	8 (29)	7 (44)	0.340
2nd	18 (41)	13 (46)	5 (31)	
3rd or beyond	11 (25)	7 (25)	4 (25)	
ECOG PS prior to pneumonitis **				
0 or 1	34 (77)	23 (82)	11 (69)	0.456
2–4	10 (23)	5 (18)	5 (31)	
ECOG PS after improving pneumonitis				
0 or 1		5 (18)		
2–4		23 (82)		
Patients characteristics after pneumonitis				
PS worsening		19 (68)		
Chemotherapy after pneumonitis		13 (46)		
Radiographic patterns of pneumonitis *				
AIP pattern	22 (50)	9 (32)	13 (81)	0.004
NSIP pattern	6 (14)	5 (18)	1 (6)	
OP pattern	2 (5)	1 (4)	1 (6)	
HP pattern	4 (9)	4 (14)	0 (0)	
Unclassifiable pattern	10 (22)	9 (32)	2 (13)	
Drug associated with pneumonitis *				
Molecular-targeted therapy (TKIs)	11 (25)	5 (18)	6 (38)	0.169
Gefitinib	8	3	5	
Erlotinib	2	1	1	
Crizotinib	1	1	0	
Cytotoxic agents ***	32 (73)	22 (78)	10 (62)	
Containing pemetrexed	4	3	1	
Containing docetaxel	6	6	0	
Containing paclitaxel	7	5	2	
Containing vinorelbine	6	4	1	
Containing gemcitabine	7	5	2	
Containing S1	4	0	4	
Containing etoposide	1	0	1	
Immune checkpoint inhibitors	1 (2)	1 (4)	0 (0)	

ILD, interstitial lung disease; ECOG PS, Eastern Cooperative Oncology Group Performance Status; AIP, acute interstitial pneumonia; NSIP, non-specific interstitial pneumonia; OP, organizing pneumonia; HP, hypersensitivity pneumonitis; TKIs, tyrosine kinase inhibitors

* Patients who received 1st-line and 2nd-/3rd- or beyond chemotherapy, patients with AIP pattern and non-AIP pattern, and patients who received molecular-targeted therapy and cytotoxic/immunotherapy were compared using Fisher’s exact test.

** Compared with PS at the diagnosis of lung cancer, two patients had improved PS and two patients had worse PS.

*** Two patients received gemcitabine plus vinorelbine, and one paclitaxel plus gemcitabine therapy.

The PS was poor and worsened after improving pneumonitis in most survivors (82% and 62%, respectively), and over half of the survivors (54%) did not undergo systemic anti-cancer therapy after pneumonitis onset. The survival time of patients after pneumonitis onset is summarised in Fig 2C and Table 5. The median survival time was 3.5 (95% CI, 2.3–7.2) months. Findings from univariate analyses indicated that longer survival time was significantly associated with good PS after improving pneumonitis (P = 0.004).

**Table 5:** Analyses of survival time after pneumonitis during systemic anti-cancer therapy (N = 28*)

Characteristics	No. of patients (%)	Overall survival time	P-value
	(N = 28)	(months)	
Age			0.8160.816
≥75 years	5 (18)	3.6	
<75 years	23 (82)	3.5	
Sex			0.7620.762
male	25 (11)	3.5	
female	3 (89)	7.2	
Smoking status			0.6860.686
never	3 (11)	3.4	
current or former-smoker	25 (89)	2.0	
ECOG PS prior to pneumonitis			0.5600.560
0 or 1	21 (75)	3.4	
2–4	7 (25)	3.8	
ECOG PS after improving pneumonitis			0.0040.004
0 or 1	5 (18)	28.5	
2–4	23 (82)	2.7	
Stage at the diagnosis of lung cancer			0.9500.950
IIIB	9 (32)	7.0	
IV	19 (68)	3.5	
Pneumonitis grade			0.3100.310
3 or 4	23 (23)	2.7	
2	5 (77)	8.8	
Pre-existing ILD			0.0750.075
yes	6 (21)	2.3	
no	22 (79)	3.6	

ECOG PS, Eastern Cooperative Oncology Group Performance Status; ILD, interstitial lung disease

* Only survivors from pneumonitis.

### Subgroup analysis of NSCLC patients with pre-existing ILD

We conducted a subgroup analysis of 69 patients with pre-existing ILD. Of these, 22 were diagnosed with non-UIP pattern, including two pathologically proven cases. Three had ILD associated with collagen vascular disease, and 3 with asbestosis. The OS was not significantly different between patients with UIP pattern and non-UIP patterns (median [95% CI] OS, 9.4 [6.9–14.0] months versus 11.1 (5.8–18.5) months, respectively; p = 0.672). Furthermore, the occurrence of pneumonitis was not significantly different between these two groups (6 out of 47 versus 5 out of 22, respectively; p = 0.303). The tumor response of first-line therapy was not significantly different between patients with and without pre-existing ILD (22/69 vs 258/701, P = 0.413)

## Discussion

To the best of our knowledge, this study is the first to report on the poor prognosis after the onset of pneumonitis and the adverse impact of pneumonitis on survival in patients with advanced NSCLC. We also identified some important characteristics of pneumonitis and confirmed the high mortality rate of pneumonitis patients in this Japanese cohort.

Our results indicated that 6% of patients with advanced NSCLC developed pneumonitis. This incidence rate is similar to that reported in a study of Japanese (7.2%) lung cancer patients, though that study included patients with small-cell lung cancer and early stage lung cancer [3]. Additionally, our study showed that 16% of advanced NSCLC patients with pre-existing ILD developed pneumonitis. Further, pre-existing ILD was found to be a risk factor of pneumonitis. Similarly, previous studies reported that approximately 20% of patients with pre-existing ILD developed pneumonitis, and that pre-existing ILD is the most significant risk factor [3, 20–24]. Based on these findings, pneumonitis seems to be a relatively common event in patients with advanced NSCLC. It is therefore necessary to monitor NSCLC patients with pre-existing ILD very closely as they are at high risk of developing pneumonitis.

This study showed other important characteristics of pneumonitis. In this study, approximately 70% of pneumonitis cases occurred during the second or subsequent lines of systemic anti-cancer therapy. Additionally, patients tended to develop pneumonitis early on during the regimen that triggered pneumonitis. We observed that most patients (73%) developed pneumonitis during the first or second cycles of therapy, with an observed median time of 1.3 months between the start of the systemic anti-cancer therapy regimen that triggered pneumonitis and the onset of pneumonitis. From these findings, physicians had difficulty predicting the onset of pneumonitis according to the line of systemic anti-cancer therapy. However, increased physician awareness of pneumonitis risk factors, and particularly careful surveillance during the period immediately after the start of systemic anti-cancer therapy, are needed because of the increased risk during this time.

We clearly demonstrated that pneumonitis has an adverse impact on survival in patients with advanced NSCLC. Pneumonitis was an independent predictive factor for OS according to multivariate analysis. Additionally, pneumonitis was significantly associated with shorter OS in those patients without pre-existing ILD, which was found to be a risk factor of pneumonitis and a strong negative predictive factor of OS. These results suggest that the management of advanced NSCLC patients with pneumonitis, including prevention, detection, and treatment of pneumonitis, is an area where there is further scope for improvement. When we consider that pneumonitis is a prognostic factor and a common event in patients with advanced NSCLC, it is apparent that further studies focusing on pneumonitis would contribute to improving the prognosis of NSCLC patients as a whole.

As shown, the early mortality rate of pneumonitis was 36%. A previous study on 49 patients, who developed drug-induced acute lung injury (73% received chemotherapeutic/anti-inflammatory agents) at the intensive care unit (ICU), reported a higher ICU mortality rate (35%) than that of non-drug-induced acute lung injury [4]. Another small study reported that about 30% of patients with pneumonitis suffered an early death [3]. These findings are similar to our results and indicate the high mortality rate of pneumonitis. Additionally, though eight patients with severe pneumonitis (grade 4 or 5) required mechanical ventilation in this study, seven of them died. Consistently, extremely high mortality rates were reported in previous studies on lung cancer patients with acute respiratory failure requiring ICU admission and mechanical ventilation [25–28]. Therefore, we should present these severe mortality data to the patients and family members to help in the shared decision-making related to the management of acute respiratory failure, including the use of mechanical ventilation.

This study also showed that the survival time was extremely poor in survivors of pneumonitis. One possible explanation for this poor prognosis is the worsening of the PS after pneumonitis. Among survivors of pneumonitis, PS was poor and this worsened after improving pneumonitis (82% and 62%, respectively). Additionally, over half of the survivors (54%) did not receive systemic anti-cancer therapy after pneumonitis. Furthermore, subgroup analyses of survivors indicated that a good PS after improvement of pneumonitis, but not other clinical factors prior to the onset of pneumonitis, was the only significant predictive factor of longer OS. In general, PS is the most important factor for evaluating the prognosis and appropriateness of systemic anti-cancer therapy in patients with advanced NSCLC. The benefits of systemic anti-cancer therapy are speculative and the prognosis is poor in patients with poor PS [29–33]. For the management of patients with pneumonitis, we should consider the prognosis and potential worsening of the patient’s condition after pneumonitis in order to make the most appropriate shared decisions [34, 35].

Pre-existing ILD was an independent predictive factor of OS in all patients, although the response rates to systemic anti-cancer therapy were not significantly different between patients with and without pre-existing ILD. These were similar to the results of previous reports [36–38]. In our study, we performed a subgroup analysis of 69 patients with pre-existing ILD. The occurrence of pneumonitis and OS were not significantly different between patients with UIP and non-UIP patterns. A previous study showed that according to CT findings, the UIP pattern was a risk factor of pneumonitis [12], while other studies did not find a significant association between these factors [39, 40]. Additionally, previous reports also indicated that the CT pattern was not associated with the survival [41, 42]. Larger studies are needed to investigate these associations in patients with pre-existing ILD.

**Fig 3:**
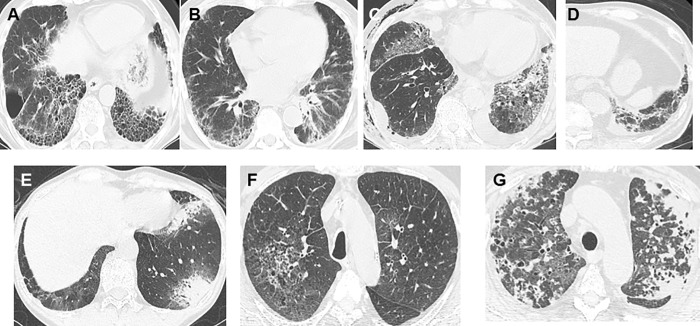
Pre-existing interstitial lung disease (ILD) showing subpleural distribution, honeycomb cysts, and bronchiectasis (usual interstitial pneumonia [UIP] pattern) (Fig 3A). Pre-existing ILD showing patchy ground-glass opacity with reticulation, traction bronchiectasis and bronchovascular bundle thickening (non-UIP pattern) (Fig 3B). Drug-related pneumonitis showing new diffuse ground-glass opacities, consolidation and traction bronchiectasis as well as pleural effusion, indicative of AIP pattern (Fig 3C). Drug-related pneumonitis showing new ground-glass opacities and bronchovascular bundle thickening, indicative of on-specific interstitial pneumonia (NSIP) pattern (Fig 3D). Drug-related pneumonitis showing new ground-glass opacities and consolidations with multifocal distribution, indicative of OP pattern (Fig 3E). Drug-related pneumonitis showing new diffuse faint ground glass opacities, indicative of HP pattern (Fig 3F). Drug-related pneumonitis showing diffuse new ground-glass opacities. Since the CT showed multiple consolidation (lung cancer) in both lungs, we could not classify the type of drug-related pneumonitis (unclassifiable pneumonitis) (Fig 3G).

This study had some limitations. First, we recognize the retrospective, single-center study design and the limited number of patients in the study group as important limitations. However, this is one of the largest studies of pneumonitis patients. Previous studies reported that Japanese may have an increased genetic susceptibility to ILD [21, 43]. Thus, the results of this study may be different in other ethnic groups. Second, the diagnosis of pneumonitis was based on physicians in charge without predetermined protocol because this study was conducted in a historical fashion. This limitation remains a challenge because attributing causality is an unavoidable problem in drug-related studies. From this aspect, in patients with pre-existing ILD, we could not separate cases of pneumonitis into acute exacerbation of pre-existing ILD and drug-related pneumonitis. The problem is the absence of gold-standard diagnostic tests and the definition of the time elapsed between the drug administration and the onset of pneumonitis. However, we carried out several tests, including bronchoscopic tests with BAL, to exclude other causes. Since pneumonitis is a diagnosis of exclusion, these tests are useful to exclude common causes of pulmonary infiltrates such as infection and lymphangitic spread of the cancer [11, 17]. In our study, we carried out the bronchoscopic tests with BAL in about half of our patients with pneumonitis (probable cases). In contrast, in previous studies, the diagnosis was based only on medical history, laboratory data, and CT scan images [3, 4, 12]. Therefore, we believe the diagnostic quality of pneumonitis was higher in this study. Third, the evaluation of the pattern of ILD was based only on HRCT findings in many patients (Fig 3). A recent paper demonstrated that the non-UIP pattern (inconsistent with UIP pattern) was miscategorised if the diagnosis is based solely on radiologic findings [44]. However, it was reported that in clinical practice, surgical lung biopsies were performed in only 8%–12% of patients [45]. Moreover, another recent report demonstrated that the radiologic evaluation was important to assess the survival and occurrence of pneumonitis [12]. Therefore, we evaluated these patterns in our study.

## Conclusions

We found that 6% of patients with advanced NSCLC developed pneumonitis during systemic anti-cancer therapy. The early mortality rate of pneumonitis is high, and the survival and performance status after pneumonitis is poor. Additionally, pneumonitis has an adverse impact on the OS of patients with advanced NSCLC. We should consider these data for the management of pneumonitis in patients with advanced NSCLC, and conduct future studies focusing on pneumonitis to improve the survival of these patients.

## Abbreviations

BAL: bronchoalveolar lavage
CI: confidence interval
EGFR: epidermal growth factor receptor gene
HRs: hazard ratios
ILD: interstitial lung disease
NSCLC: non-small lung cancer
OS: overall survival
OR: odds ratio
PS: performance status
